# Supplementary material for: Mismatch repair deficiency predicts response to HER2 blockade in HER2-negative breast cancer
Source: Nat Commun. 2021 May 19;12:2940. doi: 10.1038/s41467-021-23271-0 (PMC8134423; doi:10.1038/s41467-021-23271-0)
Supplement: Supplementary file 1 — Supplementary Information [file 41467_2021_23271_MOESM1_ESM.pdf]

Supplementary Materials:

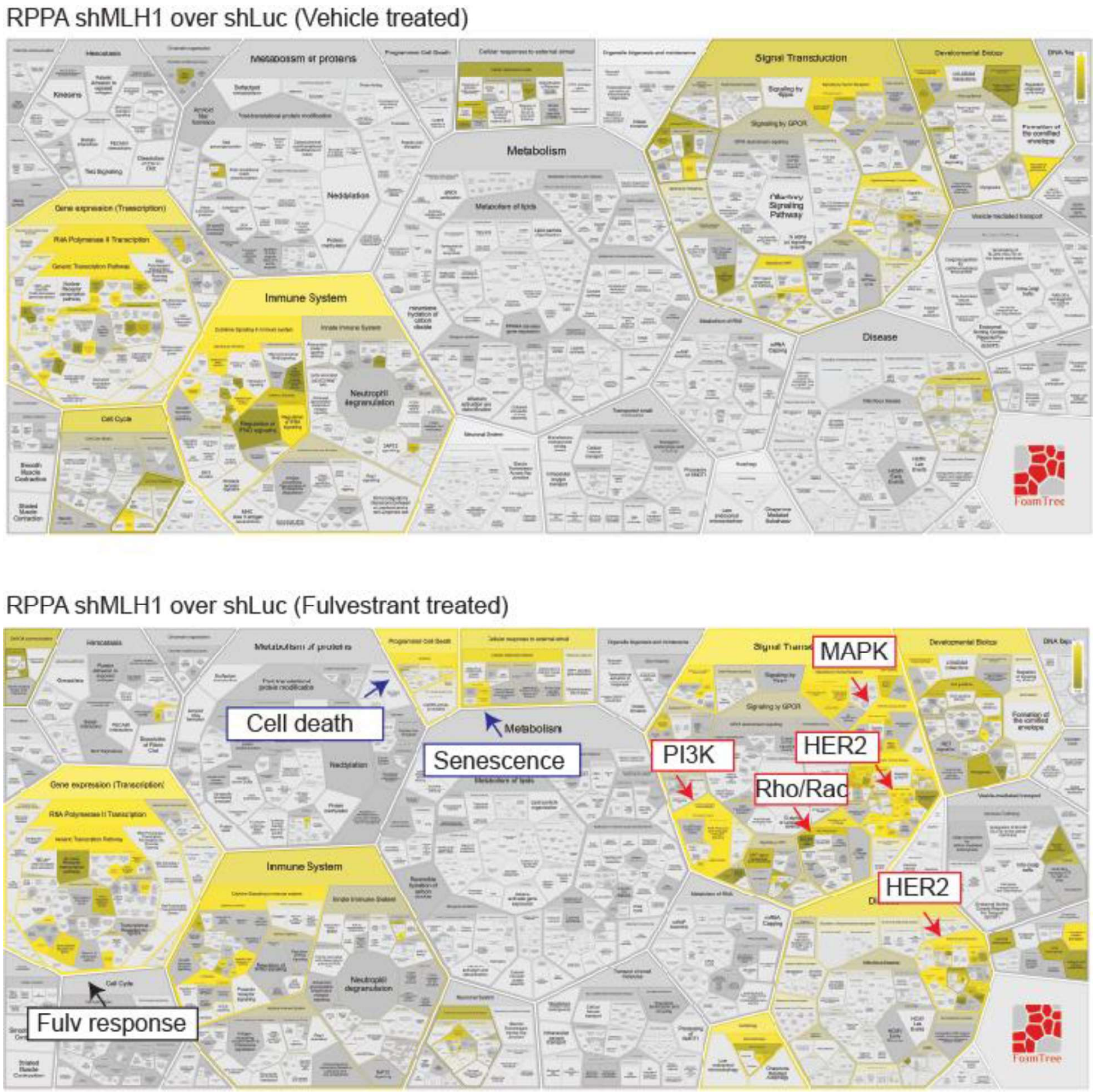

Supplementary Figure 1: ER$^+$, nominally HER2$^-$ breast cancer cells engineered to harbor stable RNAi against the MutL complex upregulate HER2 signaling pathways upon treatment with endocrine intervention, fulvestrant. Reactome analysis of RPPA data from MCF7 sh*Luc*, sh*MLH1* and sh*PMS2* cells grown +/- 100 nM fulvestrant and analyzed for proteins whose levels increase specifically in response to fulvestrant in sh*MutL* cells relative to sh*Luc*. P-value generated using two-tailed Student's t-test and corrected for multiple comparison using Bonferroni. All proteins with $p<0.05$ after adjustment for multiple comparison were used in Reactome analysis. Four replicates assayed per cell line per treatment. Source data provided with paper.

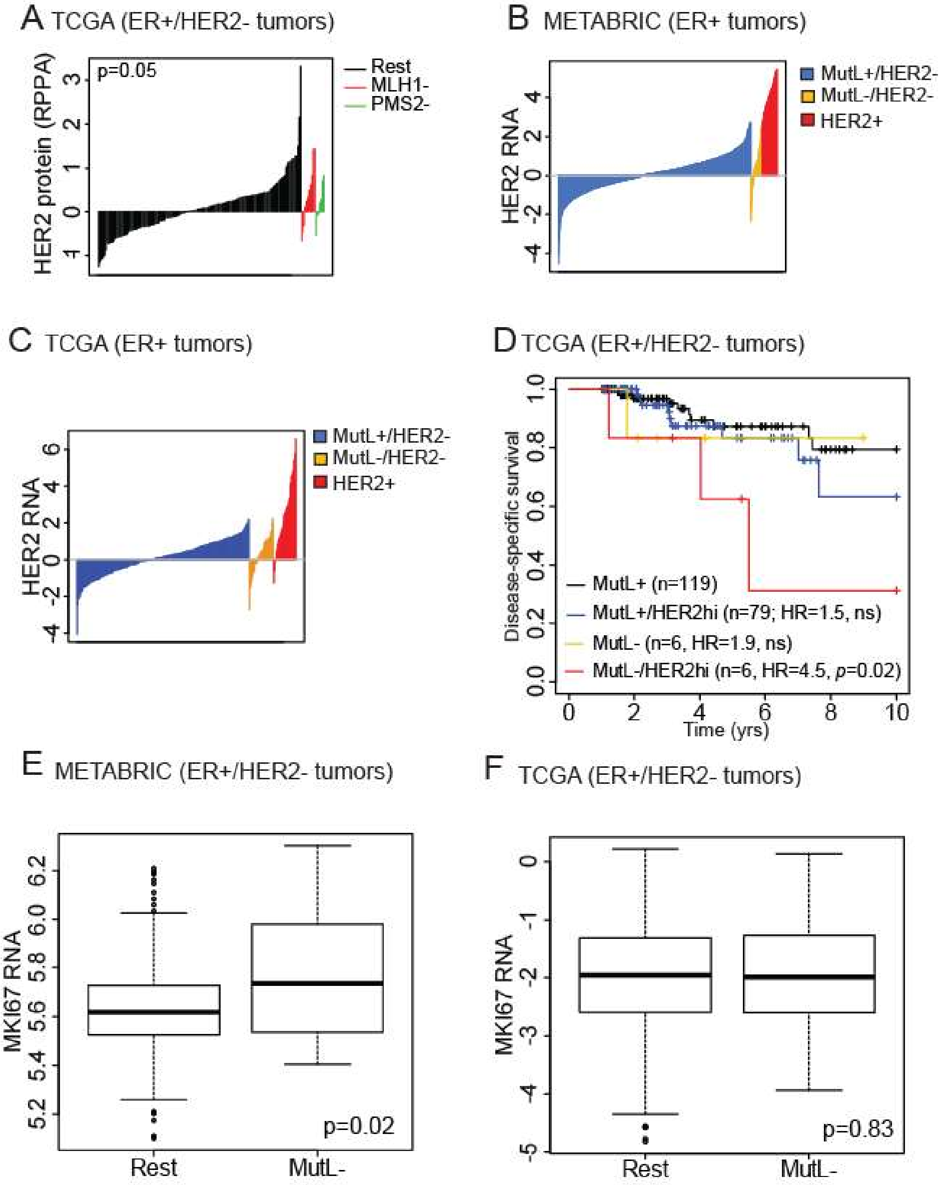

Supplementary Figure 2: ER^+, nominally $HER2^-$ breast cancer patients whose tumors are $MutL^-$ have relatively high levels of HER2 and significantly worse disease-specific outcomes. (A) Index plot depicting protein (RPPA) levels of HER2 in $MLH1^-$ (red) and $PMS2^-$ (green) ER^+/nominally $HER2^-$ patient tumors from TCGA. $MutL^+$ tumors shown for comparison in black. P-value generated using two-sided Pearson's chi-square test comparing proportion of tumors with positive values for HER2 vs those with negative values in each subgroup. (B-C) Index plots depicting RNA (microarray) levels of *HER2* in $MutL^-$ (gold) and $MutL^+$ (blue) ER^+/nominally $HER2^-$ patient tumors from METABRIC (B) and TCGA (C). $HER2^+$ (or amplified) patient tumors (red) are included to provide context. Supports data in Fig 1A. (D) Kaplan-Meier survival curves of indicated groups of patients from TCGA demonstrating differences in disease-specific survival. Cox Regression analysis determined p-values and hazard ratios. Supports data in Fig 1B. (E-F) Boxplots demonstrating comparable levels of gene expression of the proliferation marker, Ki67 in two independent datasets (METABRIC, E and TCGA, F) between $MutL^-$ (n=23 for METABRIC and n=31 for TCGA) and $MutL^+$ (n=594 for METABRIC and n=287 for TCGA) ER^+/$HER2^-$ patient tumors. Two-sided Wilcoxon Rank sum test determined p-values. Box plots show median, quartiles, minima and maxima, and outliers at 1.5xIQR. Source data provided with paper.

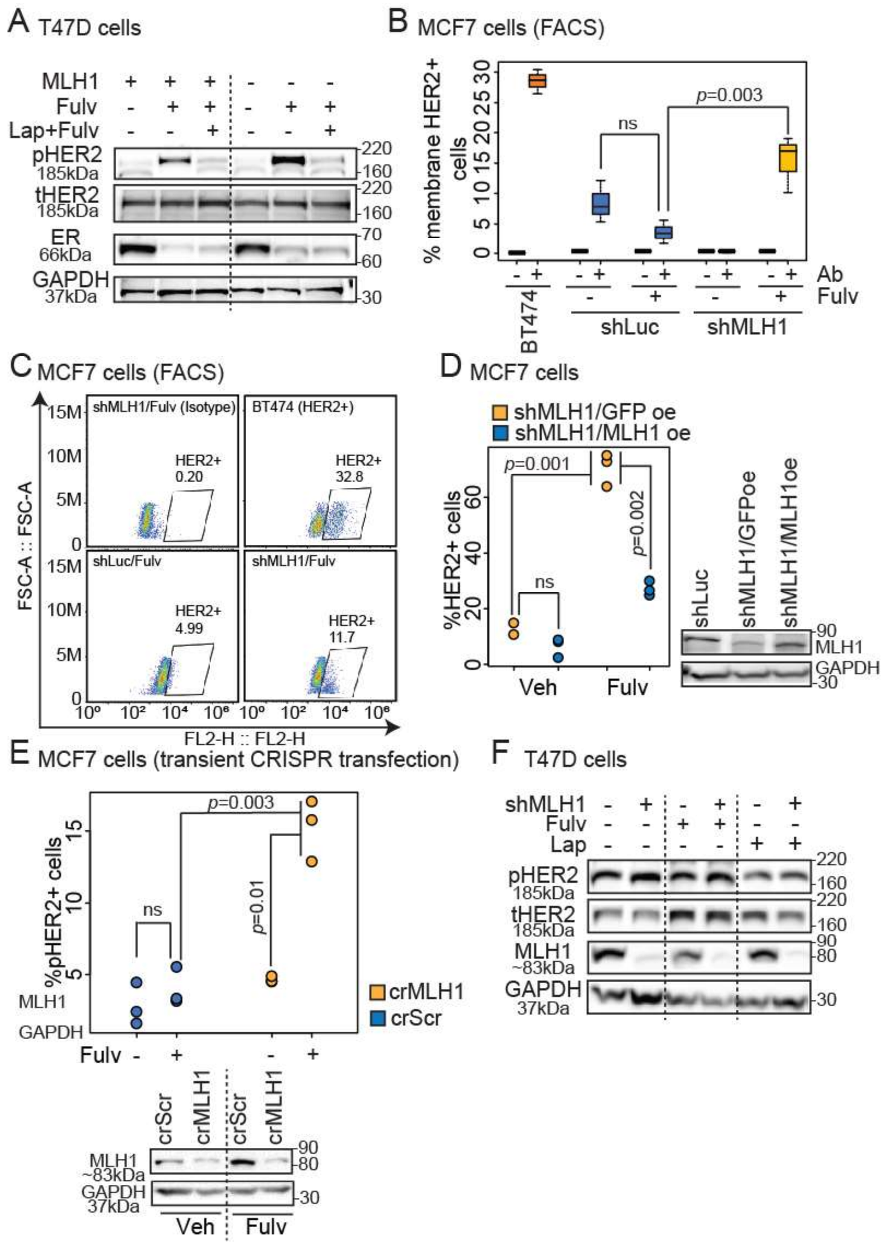

Supplementary Figure 3: MLH1 loss in ER^{+}, nominally $HER2^{-}$ breast cancer cells upregulates membrane-bound HER2 upon endocrine treatment. (A) Western blot demonstrating increased pHER2 in sh*Luc* and sh*MLH1* T47D cells treated with vehicle, fulvestrant (Fulv) or a combination of lapatinib, a HER inhibitor, and fulvestrant (Lap+Fulv). Supports data in Fig 2A. (B-C) Boxplots representing percent cells with membrane bound HER2 detected by FACS in sh*Luc* and sh*MLH1* MCF7 cells treated with or without fulvestrant (Fulv). Each group has an isotype control (Ab) and BT474, HER2-amplied cells are included as positive control. Accompanying plots demonstrate gating strategy (C). Box plots show median, quartiles, minima and maxima. Supports data in Fig 2B. (D-E) Quantification of HER2 positivity using immunofluorescence with a total HER2 antibody (D) and a pHER2 antibody (E) in MCF7 cells with re-expression of sh-resistant wildtype *MLH1* cDNA (D) or transient transfection with a CRISPR plasmid with sgRNA against *MLH1* (E), represented as strip charts. Accompanying Western blots validating MLH1 re-expression and knockdown respectively. (F) Western blot demonstrating MLH1 levels in T47D cells treated with lapatinib (Lap) to inhibit HER2 signaling. ns, not significant. For panels B-E, three biological replicates were assayed in each group for each cell line. For panel A, two independent replicates were assayed and for panel F, four independent replicates. Two-sided Student's t-test determined p-values.

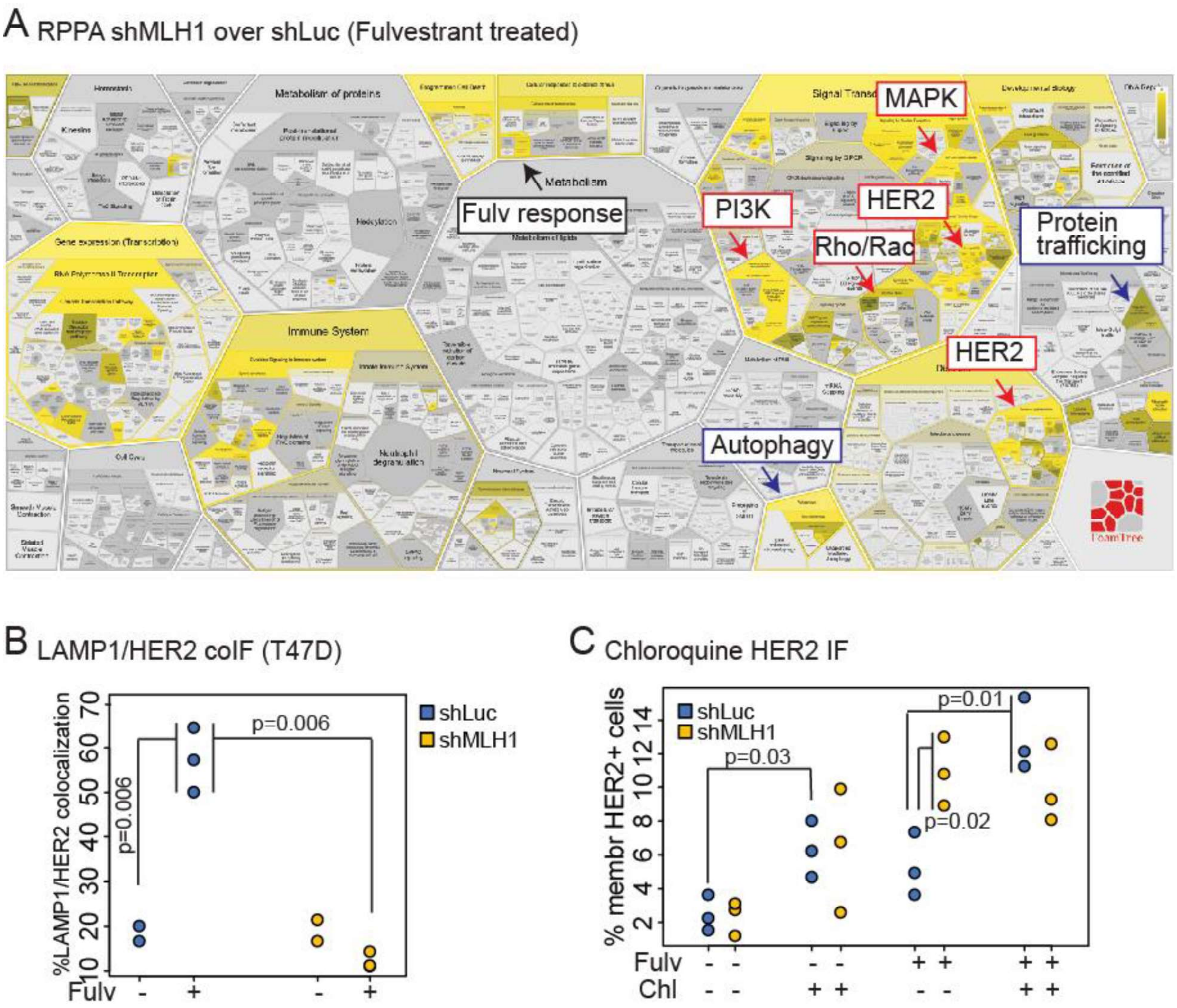

Supplementary Figure 4: HER2 is protected from lysosomal trafficking in ER^+ $MLH1^-$ breast cancer cells. (A-B) Reactome analysis of RPPA data demonstrating enrichment for protein trafficking and autophagy pathways in $MutL^-$ MCF7 cells. Corresponding RNAseq data in Fig 3A. (B-C) Strip charts representing quantification of colocalization of LAMP1 and HER2 (B) and membrane HER2 (C) immunofluorescent signaling in shLuc and shMLH1 T47D cells before and after endocrine therapy in the form of 100 nM fulvestrant (Fulv), and with or without treatment with chloroquine, an autophagy inhibitor (C). For panels B and C, three biological replicates were included in each group for each cell line. Corresponding data in MCF7 cells in Figs 3B-C. Two-sided Student's t-test determined all p-values. Source data available with paper.

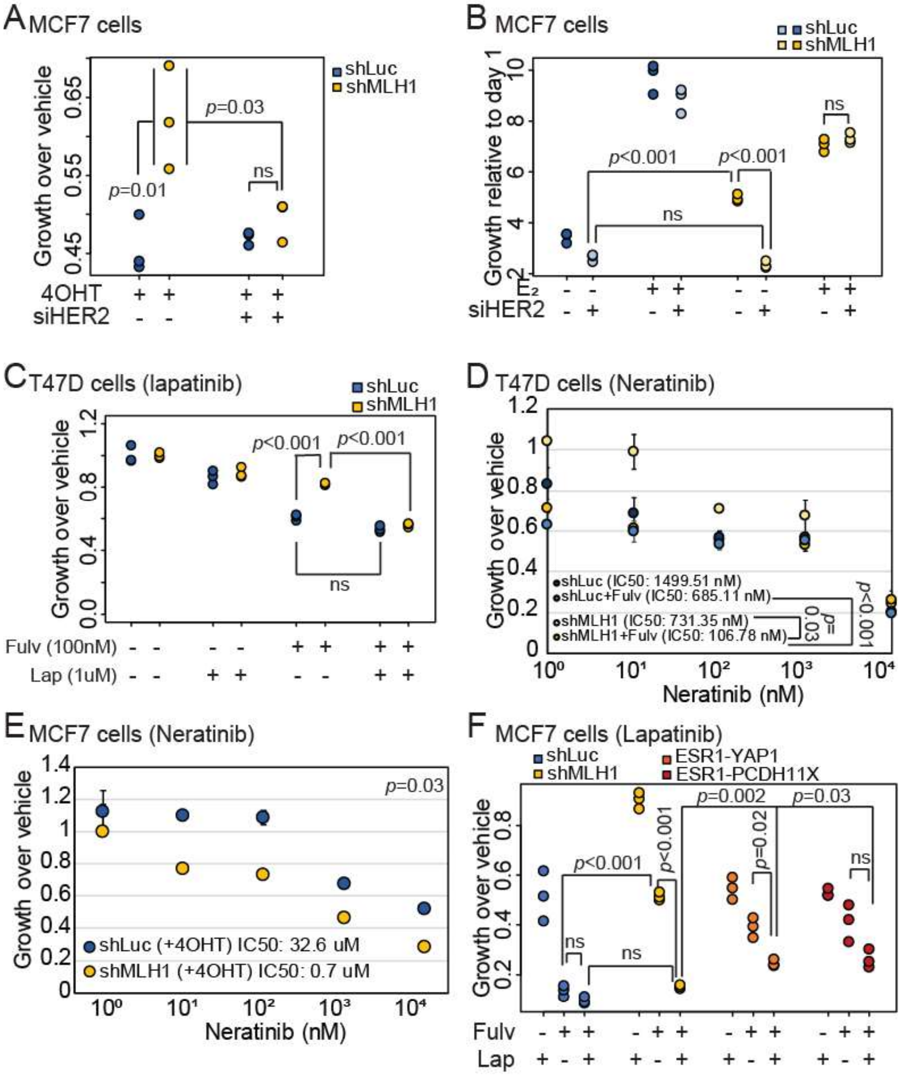

Supplementary Figure 5: HER2 is required for endocrine therapy resistant growth of ER^+ $MLH1^-$ breast cancer cells. (A-B) Bar graphs representing relative growth of MCF7 sh*Luc* and sh*MLH1* cells transiently transfected with either scrambled siRNA or siRNA against *HER2*, and then treated with tamoxifen (4-OHT, A) or grown in charcoal stripped serum and deprived of estrogen (E_2, B). For panel B, shLuc vs shMLH1 estrogen deprived cells, p=0.0008, and for shMLH1 siScr vs siHER2 estrogen deprived cells, p=2.4e-05. Western blotting validating knockdown and response to fulvestrant presented in Fig 4A-B. (C) Strip chart demonstrating increased sensitivity of T47D sh*MLH1* cells to a combination of fulvestrant (Fulv) and lapatinib (Lap). For shLuc vs shMLH1 fulvestrant treated, p=0.0009 and for shMLH1 fulvestrant vs fulvestrant+lapatinib treated, p=3.15e-05. Supports data presented in Fig 4C. (D-E) Dose response curves of sh*Luc* and sh*MLH1* T47D (D) and MCF7 (E) cells treated with neratinib in combination with fulvestrant (Fulv, D) or tamoxifen (Tam, E). IC50 values were calculated and differences in IC50 from three independent experiments was statistically compared. For shLuc vs shMLH1 fulvestrant treated cells, p=0.0001. Circles represent mean relative growth and error bars the standard deviation. Supports data presented in Fig 4D. (F) Strip chart demonstrating specificity of lapatinib response in $MutL^-$ cells when compared to non-mismatch repair related mechanisms of endocrine therapy resistance (*ESR1* fusions). For shLuc vs shMLH1 fulvestrant treated cells, p=4.46e-05, and for shMLH1 fulvestrant vs fulvestrant+lapatinib treated cells, p=7.37e-05. Three biological replicates included in each group for each cell line. Two-sided Student's t-test determined all p-values. Source data available with paper.

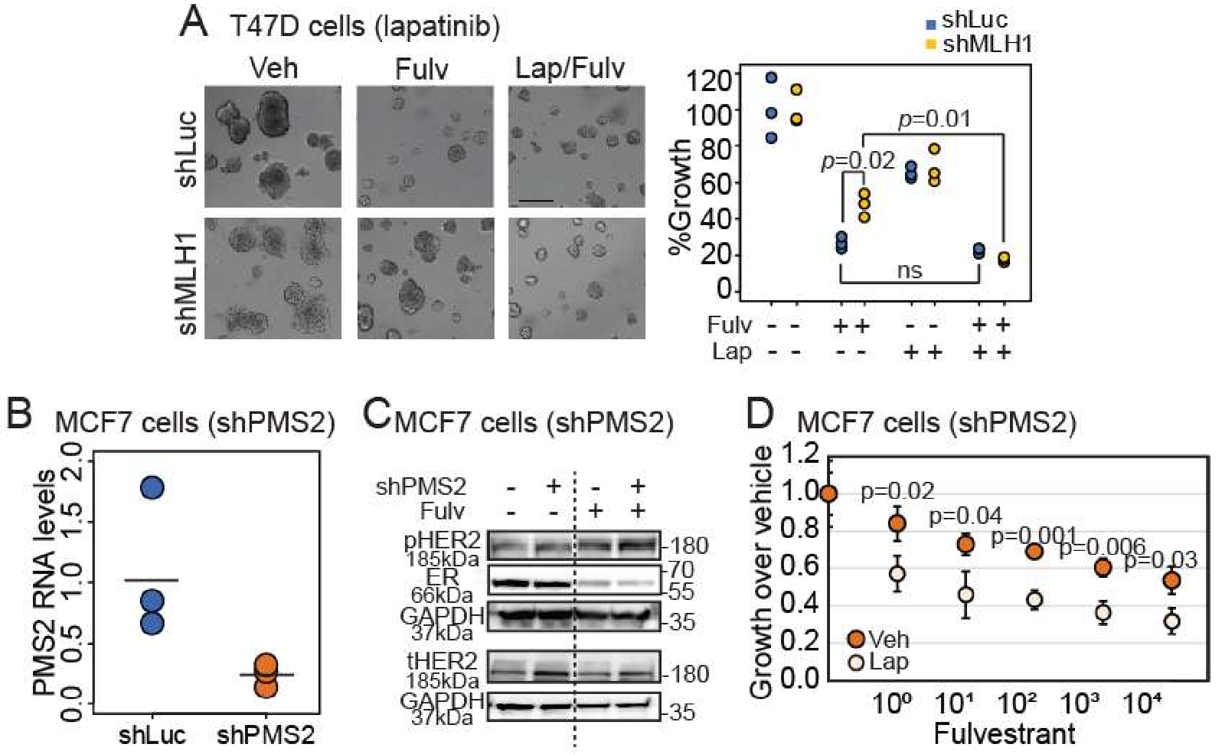

Supplementary Figure 6: HER2 is required for endocrine therapy resistant growth of ER^{+} $MLH1^{-}$ breast cancer cells. (A) 3D growth of sh*Luc* and sh*MLH1* T47D cells in Matrigel shown with representative photomicrographs and accompanying quantification. Scale bars represent 50µ. Supports data presented in Fig 4E. (B-D) Knockdown of *PMS2* in MCF7 cells validated by qRT-PCR (B) with Western blot showing increased HER2 activation in sh*PMS2* cells (C) and increased sensitivity to the combination of HER inhibition (lapatinib, Lap) and endocrine therapy (fulvestrant, Fulv) (D). Two-sided Student's t-test determined all p-values. For panels A, B and D, three biological replicates were included in each group for each cell line. Source data available with paper.

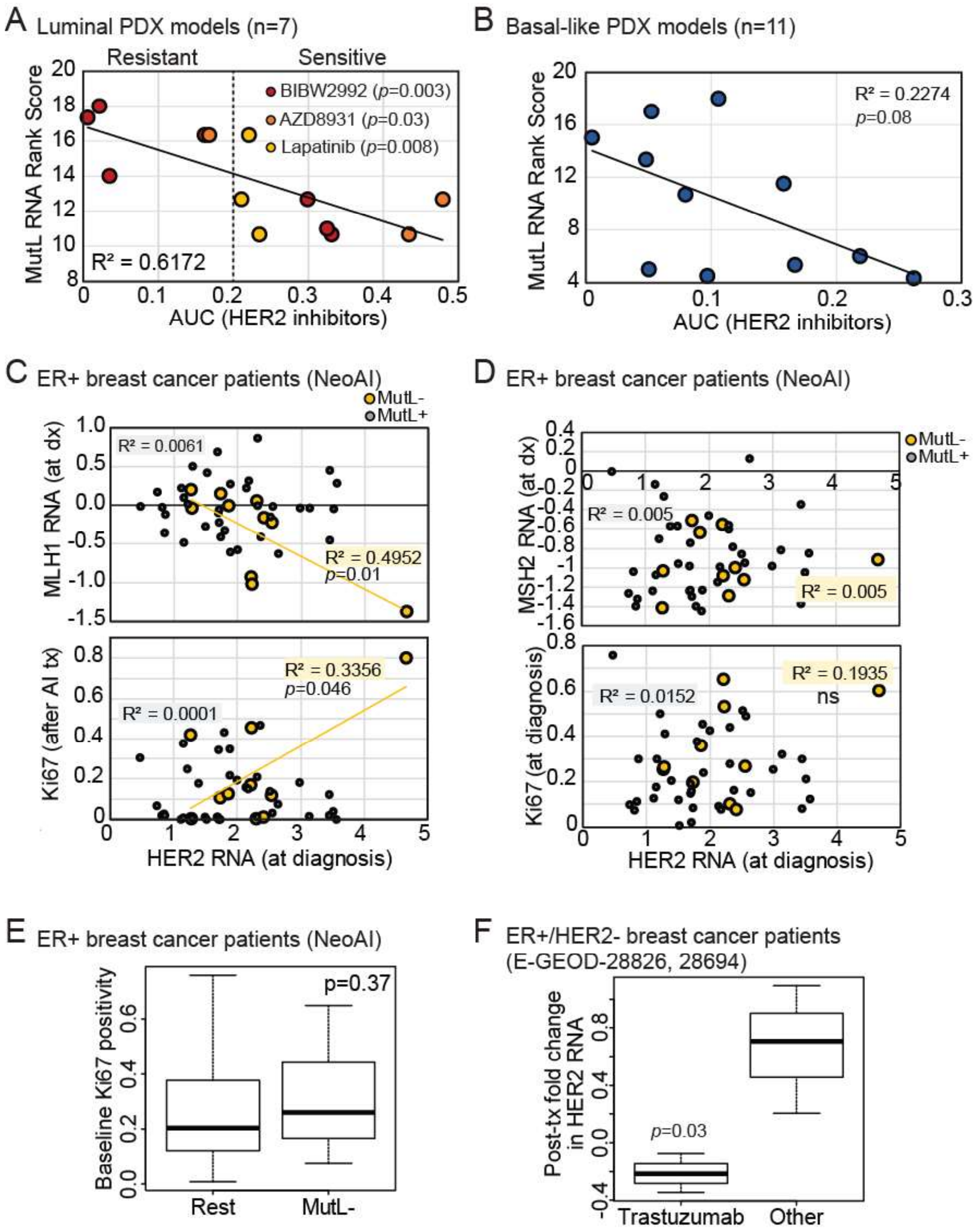

Supplementary Figure 7: MLH1 loss predicts sensitivity to HER2 inhibitors in endocrine therapy resistant ER^+, nominally $HER2^-$ breast cancer cells *in vivo* and in patient tumors. (A-B) Regression analysis depicting lack of significant correlation between *MLH1* and *PMS2* RNA levels, and sensitivity to HER2 inhibitors in luminal (A) and basal-like (B) PDX tumors grown *in vivo*. Supports data in Fig 5D-E. (C-D) Regression analysis of correlation between *MLH1* RNA levels at diagnosis (top) and Ki67 levels after treatment with endocrine interventions (bottom) (C) and *MSH2* RNA levels (top) and proliferation marker, Ki67 before endocrine treatment (bottom) (D) with *HER2* RNA levels. For all graphs, linear regression model analysis in R was used to determine R^2 and p-values. (E-F) Boxplots demonstrating comparable levels of Ki67 positivity between $MutL^-$ (n=11) and $MutL^+$ (n=37) $ER^+/HER2^-$ breast tumors from the NeoAI database (E) and downregulation of *HER2* RNA levels in response to trastuzumab (n=10), but not in response to anthracyclines or taxanes (Other, n=8, F). Supports data in Fig 5F. Box plots show median, quartiles, minima and maxima, and outliers at 1.5xIQR. Two-sided Wilcoxon Rank Sum test determined p-value. P-values were not adjusted for multiple comparisons. Source data for all panels except those dealing with Z1031 data available with paper. Access to source data from Z1031 trial available upon request with permission from Alliance Clinical Trials.

Supplementary Table 1

Name	Sequence	Catalogue Number
CRISPR MLH1 Target Sequence	Target 3: GATGGAGCGAATATTGTCCA<br>Target 2: CAGATCCAAGACAATGGCAC<br>Target 1: AGTGGTGAACCGCATCGCGG	301901110595
PMS2 Oligo Reverse	TTATCAGTTCTGAGAAATGACACCCAGGTTGG	
PMS2 Oligo Forward	CACCATGGAGCGAGCTGAGAGCTCGAGTA	
esiRNA toward HER2 Target sequence	CCATCTGCACCATTGATGTCTACATGATCATGGTCAAATGTTGGATGATTGACTCTGAATGTCG<br>GCCAAGATTCCGGGAGTTGGTGTCTGAATTCTCCCGCATGGCCAGGGACCCCCAGCGCTTTGT<br>GGTCATCCAGAATGAGGACTTGGGCCCAGCCAGTCCCTTGGACAGCACCTTCTACCGCTCACTG<br>CTGGAGGACGATGACATGGGGGACCTGGTGGATGCTGAGGAGTATCTGGTACCCCAGCAGGG<br>CTTCTTCTGTCCAGACCCTGCCCCGGGCGCTGGGGGCATGGTCCACCACAGGCACCGCAGCTC<br>ATCTACCAGGAGTGGCGGTGGGGACCTGACACTAGGGCTGGAGCCCTCTGAAGAGGAGGCCC<br>CCAGGTCTCCACTGGCACCCTCCGAAGGGGCTGGCTCCGATGTATTTGATGGTGACCTGGGAA<br>T	EHU078751-50UG
PMS2 shRNA	CCGGGGACTATGGAGTGGATCTTATCTCGAGATAAGATCCACTCCATAGTCCTTTTTG	TRCN0000425805
MLH1 shRNA	CCGGGTGTTCTTCTTTCTCTGTATTCTCGAGAATACAGAGAAAGAAGAACACTTTTTG	SHC007
Luciferase shRNA	CCGGCGCTGAGTACTTCGAAATGTCCTCGAGGACATTTCGAAGTACTCAGCGTTTTT	TRCN0000288641
