# Supplementary material for: Mismatch repair deficiency predicts response to HER2 blockade in HER2-negative breast cancer
Source: Nat Commun. 2021 May 19;12:2940. doi: 10.1038/s41467-021-23271-0 (PMC8134423; doi:10.1038/s41467-021-23271-0)
Supplement: Supplementary file 3 — Description of Additional Supplementary Files [file 41467_2021_23271_MOESM3_ESM.docx]

Description of Additional Supplementary Files

Title: Supplementary Data 1.

Description: Raw read counts from RNAseq of two independent replications of MCF7 shLuc and shMLH1 cells treated with either vehicle or 100nM fulvestrant for 4 days are presented. RNAseq was conducted on the Illumina NovaSeq platforms with paired-end 150 bp sequencing. Alignments were parsed using STAR program.
