# Supplementary material for: Mismatch repair deficiency predicts response to HER2 blockade in HER2-negative breast cancer
Source: Nat Commun. 2021 May 19;12:2940. doi: 10.1038/s41467-021-23271-0 (PMC8134423; doi:10.1038/s41467-021-23271-0)
Supplement: Supplementary file 5 — Reporting Summary [file 41467_2021_23271_MOESM5_ESM.pdf]

nature research

Corresponding author(s): Svasti Haricharan
Last updated by author(s): Apr 9, 2021

Reporting Summary

Nature Research wishes to improve the reproducibility of the work that we publish. This form provides structure for consistency and transparency in reporting. For further information on Nature Research policies, see our Editorial Policies and the Editorial Policy Checklist.

Statistics

For all statistical analyses, confirm that the following items are present in the figure legend, table legend, main text, or Methods section.

n/a	Confirmed	
☐	☒	The exact sample size (*n*) for each experimental group/condition, given as a discrete number and unit of measurement
☐	☒	A statement on whether measurements were taken from distinct samples or whether the same sample was measured repeatedly
☐	☒	The statistical test(s) used AND whether they are one- or two-sided<br>*Only common tests should be described solely by name; describe more complex techniques in the Methods section.*
☐	☒	A description of all covariates tested
☐	☒	A description of any assumptions or corrections, such as tests of normality and adjustment for multiple comparisons
☐	☒	A full description of the statistical parameters including central tendency (e.g. means) or other basic estimates (e.g. regression coefficient) AND variation (e.g. standard deviation) or associated estimates of uncertainty (e.g. confidence intervals)
☐	☒	For null hypothesis testing, the test statistic (e.g. *F*, *t*, *r*) with confidence intervals, effect sizes, degrees of freedom and *P* value noted<br>*Give P values as exact values whenever suitable.*
☒	☐	For Bayesian analysis, information on the choice of priors and Markov chain Monte Carlo settings
☒	☐	For hierarchical and complex designs, identification of the appropriate level for tests and full reporting of outcomes
☒	☐	Estimates of effect sizes (e.g. Cohen's *d*, Pearson's *r*), indicating how they were calculated

Our web collection on statistics for biologists contains articles on many of the points above.

Software and code

Policy information about availability of computer code

Data collection	No software was used
Data analysis	RNAseq data was analyzed using DESeq2 by Novogene

For manuscripts utilizing custom algorithms or software that are central to the research but not yet described in published literature, software must be made available to editors and reviewers. We strongly encourage code deposition in a community repository (e.g. GitHub). See the Nature Research guidelines for submitting code & software for further information.

Data

Policy information about availability of data

All manuscripts must include a data availability statement. This statement should provide the following information, where applicable:

- Accession codes, unique identifiers, or web links for publicly available datasets
- A list of figures that have associated raw data
- A description of any restrictions on data availability

The patient datasets analyzed during the current study are all publicly available from cBio data portal at cbioportal.org (TCGA and METABRIC), or from Gene Expression Omnibus (GEO) (https://www.ebi.ac.uk/arrayexpress/experiments/E-GEOD-28826/). Z1031 ClinicalTrials.gov Identifier: NCT00265759. Data for Z1031 samples available in dbGaP (phs000472.v2.p1). Raw readcount data from RNAseq data that support the findings of this study are available in Supplementary data. Source data are provided with this paper.

Field-specific reporting

Please select the one below that is the best fit for your research. If you are not sure, read the appropriate sections before making your selection.

- [x] Life sciences
- [ ] Behavioural & social sciences
- [ ] Ecological, evolutionary & environmental sciences

For a reference copy of the document with all sections, see nature.com/documents/nr-reporting-summary-flat.pdf

Life sciences study design

All studies must disclose on these points even when the disclosure is negative.

Sample size	Sample size for animal experiments was estimated using power.prop calculations in R.
Data exclusions	No data were excluded from the analyses
Replication	All experiments were conducted in triplicate, and each experiment was duplicated independently >2 times. All attempts were successful. Orthogonal techniques were used wherever possible to confirm phenotypic results.
Randomization	Experimental groups were randomized for all animal experiments, ensuring that the tumor volumes between groups were comparable at randomization. Similarly for cell line experiments, cells were plated and then randomized to various treatment groups. For patient datasets, groups were identified based on MLH1 and PMS2 gene expression with those tumors with mean>-1.5 standard deviation considered as MutL- and the rest MutL+. Similar classifications were used for HER2+ status.
Blinding	Investigators were not blinded to groups when data was collected but were blinded to groups when data was analyzed for immunochemistry experiments. For animal experiments, investigators were blinded to groups for data collection and analysis. For all other experiments, investigators were not blinded.

Reporting for specific materials, systems and methods

We require information from authors about some types of materials, experimental systems and methods used in many studies. Here, indicate whether each material, system or method listed is relevant to your study. If you are not sure if a list item applies to your research, read the appropriate section before selecting a response.

Materials & experimental systems

n/a	Involved in the study	
[ ]	[x]	Antibodies
[ ]	[x]	Eukaryotic cell lines
[x]	[ ]	Palaeontology and archaeology
[ ]	[x]	Animals and other organisms
[ ]	[x]	Human research participants
[x]	[ ]	Clinical data
[x]	[ ]	Dual use research of concern

Methods

n/a	Involved in the study	
[x]	[ ]	ChIP-seq
[ ]	[x]	Flow cytometry
[x]	[ ]	MRI-based neuroimaging

Antibodies

Antibodies used	Primary antibodies used include pHER2 (EMD millipore; cat# 06-229; 1:200), Ki67 (Novus Biologics, cat# NB500-170SS ,1:250). LAMP1 (proteintech, cat# 21997-1-AP, 1:750) and HER2 (Invitrogen, cat# MA5-13105, 1:1000), pHER2 Y1196 (D66B7) (Cell Signaling; cat# 6942S, 1:1000), total HER2 (Thermo Scientific; NeoMarkers; cat# MS-730-P1ABX, 1:1000), pAkt S473 (D9E) XP (Cell Signaling; cat#4060S, 1:1000), total Akt (Cell Signaling; cat#9272S, 1:1000), pS6 (S235/236) (Cell Signaling; cat# 2211S, 1:1000), total S6 (5G10) (Cell Signaling; cat# 2217S, 1:1000), MLH1 (1:2,000, Sigma-Aldrich; cat# WH0004292M2), ER clone 60C (EMD Millipore; cat# 04-820, 1:1000), and GAPDH (0411) (Santa Cruz; cat# sc-47724, 1:1000).
Validation	All primary antibodies were validated by manufacturers for the application in which they were used (i.e. pHER2, total HER2, pAkt, total Akt, pS6, total S6, ER, MLH1 and GAPDH for Western blotting and Ki67, total HER2, pHER2 (Cell Signaling), LAMP1 and MLH1 for immunocytochemistry). Further, the pHER2, total HER2, pAkt, total Akt, pS6 and total S6 antibodies were validated in our lab using lapatinib as a HER inhibitor, and siRNA against HER2, in Western blots and in immunocytochemistry. ER was validated using fulvestrant as a degrader of ER for Western blots in our hands. LAMP1 was validated for immunocytochemistry assays by adding chloroquine as an inducer of autophagy for immunofluorescence experiments. Finally MLH1 was validated for immunocytochemistry and Western blots using cells with stable shMLH1.

Eukaryotic cell lines

Policy information about cell lines

Cell line source(s)	MCF7 and T47D cells were both obtained from ATCC
Authentication	Cell lines are authenticated annually by STR analysis provided by the SBP Genomics core
Mycoplasma contamination	All cells are negative for mycoplasma and all cell lines in use are tested for mycoplasma contamination every 6 months using the Lonza Mycoalert Plus Kit (cat# LT07-710)
Commonly misidentified lines (See ICLAC register)	There are no commonly misidentified lines used in this study

Animals and other organisms

Policy information about studies involving animals; ARRIVE guidelines recommended for reporting animal research

Laboratory animals	Mice for the MCF7 experiments were 4- to 6-week athymic nu/nu female mice (Envigo or the SBP animal facility). For WHIM20 PDX experiments, 6- to 8-week female SCID/Bg mice were purchased from Jackson laboratory.
Wild animals	No wild animals were used in this study
Field-collected samples	No field-collected samples were used in this study
Ethics oversight	SBP and BCM IACUC boards approved all experimental procedures

Note that full information on the approval of the study protocol must also be provided in the manuscript.

Human research participants

Policy information about studies involving human research participants

Population characteristics	Gene expression data was obtained from publicly available datasets (TCGA, METABRIC, Z1031) with either microarray or RNAseq analysis of ER+/HER2- breast tumors from women diagnosed with breast cancer.
Recruitment	Participants were recruited as per each studies outlined protocols.
Ethics oversight	All studies were performed after obtaining IRB approvals from their respective institutions.

Note that full information on the approval of the study protocol must also be provided in the manuscript.

Flow Cytometry

Plots

Confirm that:

- [x] The axis labels state the marker and fluorochrome used (e.g. CD4-FITC).
- [x] The axis scales are clearly visible. Include numbers along axes only for bottom left plot of group (a 'group' is an analysis of identical markers).
- [x] All plots are contour plots with outliers or pseudocolor plots.
- [x] A numerical value for number of cells or percentage (with statistics) is provided.

Methodology

Sample preparation	Cells were harvested and washed with chilled PBS. On ice, cells were blocked with 5% Goat serum in PBS for 30 minutes. Cells were then incubated for 2hrs at 4°C in 1% Goat Serum in PBS at 1:50 antibody dilution, washed, and resuspended in 300uL of 5% Goat Serum in PBS and run.
Instrument	BD Accuri C6
Software	FlowJo software with default settings was used to analyze the data.
Cell population abundance	Live cells were at 80-90% abundant post FSC/SSC gating, and 60% of the live cell population were determined to be singlets based on FSC-Area vs Height. 0-30% of the singlet population was HER2+ based on FL2 positivity with gates set using a HER2 amplified cell line (BT-474) as positive control and the isotype control for each cell line as negative control.

Gating strategy	Preliminary FSC/SSC gates were set in the starting cell population to restrict analysis to live cells by gating out cells with low fluorescence. FSC-A vs FSC-H axes were used to gate out multiplets by removing scattered fluorescent cells. Finally the FL2 x-axis was used to identify the cell population that was HER2+ using controls to set gates.

☒ Tick this box to confirm that a figure exemplifying the gating strategy is provided in the Supplementary Information.
